# Moving toward precision communication in pediatric oncology clinical trials: An outline of a conceptual framework to support caregiver decision-making

**DOI:** 10.1007/s00520-026-11048-4

**Published:** 2026-08-03

**Authors:** Ariane Levesque, George Blackall, Sydney A. Axson, Lauren J. Van Scoy, Giselle Saulnier Sholler

**Affiliations:** 1 Department of Pediatrics, Penn State College of Medicine, Hershey, PA USA; 2 Department of Neuroscience and Experimental Therapeutics, Penn State College of Medicine, Hershey, PA USA; 3 Ross and Carol Nese College of Nursing, Penn State University, University Park, PA USA; 4 Rock Ethics Institute, Penn State University, University Park, PA USA; 5 Department of Medicine, Penn State College of Medicine, Hershey, PA USA

**Keywords:** Pediatric oncology, Clinical trials, Caregiver decision-making, Psychosocial burden, Communication

## Abstract

Pediatric oncology clinical trials (POCTs) are the cornerstone of therapeutic progress in childhood cancer. Yet, poor accrual and retention remain persistent challenges contributing to early termination of POCTs, limiting the advancement of novel therapies. These challenges reflect a convergence of system, protocol, clinician, and family-level factors, among which caregivers’ psychosocial decision-making processes represent a potentially modifiable but underexplored contributor. Caregivers making POCT-related decisions must navigate complex medical information under conditions of heightened emotional distress and uncertainty. Psychosocial factors such as distress, comprehension, trust, values, and social context are known to influence decision-making. However, the field still lacks an integrated framework to explain how these factors may shape decisional trajectories and outcomes throughout POCT participation. This commentary reframes POCT enrollment not as a discrete informed-consent event, but as a dynamic, psychosocial decision-making process that unfolds over time. We present an outline of a conceptual Precision Communication Framework designed to support care teams in aligning communication priorities with caregivers’ psychosocial and decisional contexts at key decision points throughout trial participation. Rather than seeking to address all causes of poor accrual or withdrawal, this framework focuses on reducing preventable decisional uncertainty and regret in contexts where psychosocial processes are central drivers of caregiver experiences. By presenting an outline of a conceptual model that would link psychosocial-decisional profiles to communication strategies to prioritize, this commentary aims to inform future empirical research and lay the groundwork for identifying communication strategies to prioritize to reduce caregiver decisional burden and promote sustained participation in POCTs.

## Introduction: Early termination of pediatric oncology clinical trials

Pediatric oncology clinical trials (POCTs) have historically allowed for dramatic improvements in the survival and quality of life of children diagnosed with cancer [1]. Despite these advances, clinical trials in oncology remain vulnerable to early termination, often attributed to poor accrual [2]. Early termination, along with initial referral and the inclusion of a child in a POCT, however, reflect a complex interplay of factors, including trial availability, funding issues, logistical issues, local and national consensus for treatment options, and family-level decision-making processes [3–5]. Many of these contributors lie outside the control of individual care teams. Others, particularly those related to caregiver decision-making under conditions of psychosocial distress [6, 7], are likely modifiable. We recognize, however, that poor accrual and retention in POCTs are driven by multiple structural, protocol-level, and logistical factors, including trial availability, eligibility restrictions, and age-related disparities in enrollment [8]. Caregiver decision-making is therefore one important, but not exclusive, determinant of participation.

Few studies have directly examined how parental decision-making processes relate to withdrawal or early termination in POCTs more broadly. However, an analysis of the Children’s Oncology Group (COG) AALL932 trial reported that, although accrual goals for the POCT were met, withdrawal was relatively high, with 22% of children nationally and 40% of children at one institution withdrawn due to “parental choice” [9]. These rates are similar to the national average on other COG trials, which are typically late-phase POCTs [9, 10]. Although limited data is available regarding withdrawal from early-phase trials, the greater uncertainty about benefits and poorer overall survival expectations in early-phase POCTs raise the concern that withdrawal rates may be even higher in this context. The authors noted that parents’ reasons for withdrawing were not well understood [9], highlighting a critical knowledge gap. Furthermore, Aristizabal et al., 2021 evidenced that higher decisional regret was associated with a lower perception of having made a voluntary decision regarding POCT enrollment [11] suggesting that parental psychosocial and decisional factors may be key to understanding why some families later choose to withdraw their child from a POCT.

Caregivers considering POCT participation must make high-stakes decisions shortly after a life-altering diagnosis, often while experiencing intense emotional distress, uncertainty, and cognitive overload [6, 7]. Evidence suggests that emotional burden directly shapes decision-making processes [12] and POCT decision-making requires understanding complex scientific information during a time of profound distress [13]. Although psychosocial factors such as stress, comprehension, trust, and social context are known to influence decision-making in various populations [14–17], the field of POCTs lacks an integrated framework that highlights how these psychosocial factors intersect with decision-making patterns to shape POCT participation. In this commentary, we do not seek to present a finalized, empirically validated framework, but rather to offer an outline of a conceptual model and associated hypotheses to guide future empirical work. Specifically, this commentary aims to (a) articulate the rationale for a precision communication approach in POCTs, (b) propose preliminary hypotheses regarding psychosocial-decisional profiles and their potential implications for communication priorities, and (c) delineate next steps for empirical work needed to define, operationalize, and validate the framework. The outline of the conceptual model we propose is intended to be adaptable across trial phases (e.g., early- and late-phase POCTs) and age groups (children and adolescents/young adults), while recognizing that goals of care, decisional priorities, and the tempo and structure of informed-consent discussions may differ across these contexts.

## Psychosocial burden and decision-making under uncertainty

Caregivers commonly describe POCT-related decisions as among the most difficult experiences of their child’s cancer trajectory [6]. Heightened anxiety, loss of control, uncertainty about prognosis, and limited health literacy are common and well-documented contributors to parents’ decision-making processes [13, 18, 19].

Research in pediatric oncology communication illustrates how caregivers must rapidly absorb high-stakes information regarding experimental therapies, risk profiles, and treatment alternatives [6]. Even under typical circumstances, such tasks require cognitive clarity. Yet during the early treatment phase, caregivers experience heightened distress and mental overload, factors that are known to interfere with deliberation and increased reliance on provider recommendations [12]. These processes likely influence not only initial treatment decisions but also caregivers’ evolving interpretations of the risks and benefits of the POCT. As such, psychosocial burden is at risk of contributing to decisional uncertainty and later regret [20, 21], even when POCT information is conveyed accurately and in good faith. Psychosocial processes related to POCT decision-making likely extend beyond the initial consent discussion and may impact how caregivers interpret, re-evaluate, and engage with POCT participation over time.

Caregiver understanding, however, is influenced not only by distress but also by health literacy and preferred language [22]. These factors are especially important in pediatric oncology, where limited health literacy and language discordance are associated with lower informed consent comprehension and where families who speak languages other than English in the United State may experience substantial barriers to fully informed participation [22, 23]. Indeed, recent studies have evidenced that limited health literacy and use of Spanish for medical communication are associated with lower informed consent comprehension, and work on language discordance shows that families who speak languages other than English face major communication barriers and feel they may receive less information [22, 23].

## Decision-making patterns and caregiver psychosocial profiles: Toward an integrated model

Current literature identifies several elements that likely influence parental consent for POCT enrollment, including comprehension of the trial, the perceived lack of treatment alternatives, interpersonal dynamics with the oncology team, and the influence of distress [24, 25 ]. However, existing literature has yet to integrate psychosocial outcomes and decision-making patterns into empirically-derived profiles that predict downstream decisional and engagement outcomes.

Caregivers likely differ in how psychosocial factors such as emotional distress, trust in providers, perceived moral obligation, coping strategies, and social support intersect with their decision-making patterns (i.e., deliberation style and risk-benefit interpretation), and these processes unfold within the broader family context that includes the child or adolescent perspectives and preferences. Distinct configurations of these factors are likely to give rise to different participation trajectories, including sustained engagement, withdrawal, or preventable decisional regret. The configurations illustrated below are presented as **illustrative hypotheses** rather than empirically validated profiles. They are meant to serve as conceptual examples of how such profiles might relate to participation trajectories, rather than profiles that are already empirically established. The specific profiles and their association with participation trajectories remain to be validated in future empirical work, which is a focus of ongoing work by our team.

### Examples of how profiles may relate to participation trajectories (illustrative hypotheses that remain to be empirically validated):

- **High distress** accompanied by **low comprehension** may lead to increased reliance on provider guidance and increased risks of decisional regret.
- **High hope** accompanied by **strong moral obligation** may drive enrollment despite uncertainty, potentially affecting later retention.
- **Low trust in provider** accompanied by **limited social support** may decrease initial enrollment or increase the risk of later withdrawal.

Empirically characterizing these profiles would enable the development of structured tools to support communication strategies that are responsive to caregivers’ evolving decision-making experiences.

## The need for a Precision Communication Framework

Precision medicine has transformed oncology by tailoring treatment to biological profiles [26]. Here, we propose that, in parallel, there is a need to advance toward precision communication by tailoring trial-related discussions to caregivers’ psychosocial and decision-making contexts. This approach builds on the reality that although clinicians often adapt their communication based on their experiences with families, only 32% of parents substantially understand early-phase POCTs, and 35% have little to no understanding of these POCTs [27]. Developing and testing an individualized communication framework offers a concrete approach to bridge the gap between expectations for high-quality informed consent and parents’ limited understanding of early-phase POCTs. Communication frameworks in the context of POCTs have previously been described as a helpful tool to ensure that discussions remain effective [28]. The intent of our Precision Communication Framework will be to help structure existing practice by highlighting how caregivers experience and process POCT-related decisions. Recent American Society of Clinical Oncology (ASC) discussions underscore that POCT enrollment is shaped by broader systemic barriers, including restrictive eligibility criteria and lower enrollment among adolescents and young ﻿adults [8]﻿. The outline of the framework proposed here is intended to address modifiable psychosocial and communication processes, not to serve as a comprehensive solution to all barriers to enrollment or retention. It is intended to complement, not replace, ongoing efforts to address other structural barriers [8].

Furthermore, our proposed outline of a conceptual Precision Communication Framework is not intended to replace existing patient- and family-centered communication models and decision-support frameworks, but rather to complement them in the specific context of POCTs. For example, the Ottawa Decision Support Framework provides critical insight into how decisional needs (e.g., decisional conflict, inadequate knowledge, unclear values) and decisional outcomes require specific types of decisional support (e.g., clinical counseling, decisional tools, decision coaching) [29]. However, although this model is undeniably useful in directing participants to appropriate decisional support resources, it offers less guidance on how clinicians might adapt moment-to-moment communication strategies in real time to support families in making decisions (e.g., speech rate, validation, proxemics, kinesics). The added value of our proposed outline of a conceptual Precision Communication Framework is that it seeks to (a) operationalize caregiver psychosocial-decisional profiles in the specific context of POCTs , (b) map these profiles to concrete communication priorities for trial-related discussions, and (c) explicitly conceptualize these mappings as dynamic over time as caregivers’ experiences and interpretations of risk and benefit evolve.

Our proposed framework will be intended to be usable across key decision-making moments, beginning after diagnosis and evolving as treatment options and trial eligibility emerge, and it is conceptualized to support shared decision-making processes among patients, caregivers, and providers. Importantly, this framework will not seek to address all contributors of poor accrual or early discontinuation, nor will it aim to prevent withdrawals driven by toxicity, logistical constraints, or protocol requirements. Instead, it will target preventable decisional uncertainty or regret in contexts where psychosocial factors are primary drivers of caregivers’ decision-making experiences. Although decisional regret is relatively low in pediatric oncology more broadly (i.e., approximately 11% of parents 1 year after diagnosis [30]), 38% of bereaved parents of a child with cancer report decisional regret and an additional 12% are unsure of whether they regret their decisions [31]. Given that overall survival rates are poor for early-phase POCTs [32], many parents in this context may be at heightened risk for decisional regret, underscoring the importance of communication approaches that aim to reduce preventable regret.

Conceptually, the framework outline we propose here is based on three interrelated components. First, it will involve the identification of caregiver psychosocial-decisional profiles, reflecting patterns of emotional distress, coping strategies, trust in providers, social support, and decision-making patterns. We anticipate that, in future empirical work, these profiles will be able to be approximated even in early POCT consent discussions through brief, validated psychosocial measures and early conversational cues and first impressions, rather than relying on an extensive prior relationship. The Precision Communication Framework will be designed to assist clinicians in identifying relevant cues in the absence of a longstanding relationship. For families with limited English proficiency, the framework will also be designed to support interpreter-mediated communication by encouraging coordination between clinicians and trained medical interpreters, with the goal of ensuring that communication strategies are adapted to the family’s language and cultural context. In this way, the framework will not only guide what clinicians prioritize in conversation but also reinforce how interpreters and clinicians can work together to deliver clearer family-centered communication. To do so, future development efforts of the framework may include interpreter-focused guidance or training to help ensure that interpreter-assisted discussions preserve the intended communication priorities, including pacing, clarification, emotional validation, and checking understanding.

Second, the framework will emphasize mapping psychosocial-decisional profiles to communication priorities. Currently, the specific composition and boundaries of the caregiver psychosocial-decisional profiles remain empirical questions, underscoring the need for research to systematically characterize how these factors may cluster for caregivers in pediatric oncology. The goal of this component will be to highlight aspects of communication that may be particularly useful to support caregivers in different psychosocial contexts. For example, once the psychosocial-decisional profiles of component 1 are identified, mappings may include:

**Table:** 

Example of possible psychosocial-decisional profiles	Example of communication strategy that may be prioritized
High distress accompanied by high cognitive load^1^	Slower pacing, emotional validation, explicit acknowledgement of uncertainty
Low trust in provider and high decisional uncertainty	Transparency, increased opportunity for questions, clarification of roles and expectations
High hope and strong perceived moral obligation	Clarification of values, normalization of uncertainty, increased opportunities for re-evaluation of decisions
Tendency to defer decision-making	Encouragement of active participation in discussions, increased pauses

Examples in this table are illustrative. Clinically relevant psychosocial-decisional profiles and communication strategies to prioritize for each profile remain to be confirmed by empirical research. ^1^Although high distress and high cognitive overload are common shortly after diagnosis, this does not lessen their importance as a target within the framework; rather, it underscores the need to routinely prioritize the possible corresponding strategies.

Third, the framework will explicitly be dynamic and will be revisited over time. Communication strategies to prioritize may shift as caregivers’ experiences, emotional states, and interpretations of risk and benefit evolve throughout POCT participation. As such, the framework will be designed to support communication not only at initial trial discussions, but also at key decision points such as treatment transitions, emerging toxicities, or consideration of continued participation.

In real-world clinical practice, the Precision Communication Framework will be designed to integrate flexibly into existing workflows rather than function as a standalone tool. Use of the framework will not require lengthy formal psychosocial screening or additional clinical encounters. Given that early POCT discussions often occur in emotionally charged settings, the implementation of the framework will be designed to be lightweight. In practice, caregiver profiles may be derived from a combination of very brief self-report items and clinicians’ attention to early conversational cues (e.g., caregivers’ questions and affects). These aspects will be easily mapped onto the framework to direct clinicians to the communication strategy that may be most relevant to prioritize for a given family in a given encounter. Prior work on communication in medical contexts has shown that concise and teachable communication models, such as stepwise protocols for delivering difficult news, can be implemented in routine practice [33]. Our proposed Precision Communication Framework is intended to share these characteristics by offering concise guidance on which communication priorities to emphasize, rather than prescribing additional lengthy encounters or rigid scripts.

The framework will be designed to be applied by oncologists, advanced practice providers, and psychosocial clinicians to guide how trial-related information is introduced and revisited across clinical encounters. In doing so, it is intended to reinforce shared decision-making by supporting communication that engages caregivers, as well as patients, collaboratively with the care team. As such, the Precision Communication Framework will function as a decision-support aid that helps clinicians anticipate psychosocial needs during POCT discussions. Importantly, the communication strategies to prioritize will be able to be revisited by repeating the brief measures and/or by tuning in to evolving conversational cues as caregivers’ experiences and needs evolve over the course of POCT participation.

This framework will not aim to prescribe what clinicians must say, but will instead highlight the communication strategies that may be most effective in supporting caregivers in different psychosocial contexts. The Precision Communication Framework will be conceptualized as a support tool that could inform communication priorities based on caregivers’ psychosocial and decisional contexts, rather than as a standardized intervention or replacement for clinician judgment.

In future work, we plan to develop this conceptual model into an empirically validated framework through three development phases:

- First, through a mixed methods study, we will systematically characterize caregiver psychosocial-decisional profiles using validated quantitative measures of psychosocial outcomes and qualitative analyses of discussions inquiring about POCT-related decision-making. These discussions will occur in the context of semi-structured interviews designed to help our team identify how factors such as distress, comprehension, and values contribute to decision-making processes and decisional outcomes regarding POCTs. We will integrate these qualitative results with the quantitative psychosocial outcome measures.
- Second, we will conduct a qualitative focus group study in which we will operationalize the profiles generated in our first phase and map them onto specific, testable communication strategies identified through stakeholder and expert feedback (i.e., clinicians, caregivers, and psychologists).
- Third, we will validate the framework by examining whether profile-informed communication strategies are associated with improved outcomes, such as caregiver understanding of the POCT, perceived voluntariness, and decisional conflict/regret compared to standard communication.

These steps will be essential to move from the outline of the proposed framework detailed in this commentary to an empirically validated and rigorously defined Precision Communication Framework.

## Ethical implications and downstream outcomes

Early termination of POCTs raises ethical concerns not only at the level of scientific efficiency, but also for families who invest time, hope, and emotional energy in trial participation [4]. When participation trajectories are marked by preventable decisional uncertainty or regret, caregivers may experience a lingering emotional burden that extends well beyond the duration of the POCT.

Optimizing psychosocial decision-making processes is therefore an ethical imperative. A psychosocially attuned communication approach has the potential to ensure that participation, and withdrawal when it occurs, is grounded in informed reflection rather than avoidable distress or misunderstanding. While such an approach cannot address all drivers of early termination, it represents a meaningful step toward supporting caregiver well-being and decision-making experiences over time.

## Conclusion

POCT-related decision-making occurs in a context of emotional, cognitive, and relational complexity. Although communication in clinical care is generally individualized and responsive to the needs of families, the field currently lacks an empirically derived framework that explicitly integrates caregivers’ psychosocial and decision-making patterns over time. In this commentary, we propose a conceptual framework that explicitly links psychosocial-decisional profiles to communication priorities over time. This outline of a conceptual framework is intended to extend existing patient- and family-centered decision-support models into the distinctive context of POCTs.

By integrating psychosocial outcomes with decision-making patterns, the proposed framework will offer a structured way to conceptualize how communication priorities might be aligned with caregivers’ evolving needs across the trajectory of POCT participation. Importantly, this framework is intended to be designed as a decision-support model, not a prescriptive intervention, and will aim to accompany, rather than replace, clinician judgment and existing communication practices.

Although the specific psychosocial-decisional profiles and corresponding communication priorities described here require empirical validation, articulating this outline of a conceptual framework represents an important step toward generating testable hypotheses and guiding future research. Advancing psychosocially-attuned communication in POCTs has the potential to reduce preventable decisional regret and promote sustained engagement in POCTs over time. By addressing one modifiable contributor to caregivers’ decision-making experiences, this conceptual framework aims to inform the identification of psychosocially-attuned communication strategies to prioritize in POCT-related discussions to support caregiver well-being while responsibly advancing pediatric oncology research.

## Data Availability

No datasets were generated or analysed during the current study.

## Abbreviation

POCT: Pediatric oncology clinical trial
